# Morphometric Analysis of Spinal Cord and Its Termination Within the Vertebral Canal: An Observational Study in the Fetuses of Third Trimester Gestational Age

**DOI:** 10.7759/cureus.30438

**Published:** 2022-10-18

**Authors:** Nand K Gupta, Hetal Vaisnani, Preeti Gupta, Achleshwar Gandotra

**Affiliations:** 1 Anatomy, Smt. B. K. Shah Medical Institute & Research Centre, Vadodara, IND; 2 Physiology, Naraina Medical College & Research Centre, Kanpur, IND

**Keywords:** vertebral canal, spinal cord, gestational age, fetuses, conus medullaris

## Abstract

Background & aim

The spinal cord is the continuation of the brain from the lower point of the medulla and the terminal portion of the developing neural tube. The spinal cord develops within the bony canal, called the vertebral canal, formed by the union of individual vertebrae in the vertebral column. Initially, the development of the length of the vertebral column and spinal cord are the same but later on undergo alterations. The growth of the vertebral column is faster than that of the spinal cord because the spinal cord appears to terminate early within the vertebral canal. To measure the length of the spinal cord and lowermost point of conus medullaris in the third trimester gestational age fetuses.

Material and methods

The present cross-section observational study was carried out on 30 fetuses collected from the museum of the Anatomy Department and Obstetrics and Gynecology Department. Before starting the study, permission and approval from the university's ethical committee were received. The dissection of fetuses includes the incision of the skin, removal of superficial and deep muscles, and a laminectomy. The meninges were cut and removed to note the vertebra level of the termination of the spinal cord. The spinal cord was taken out, and the total length of the spinal cord was measured. The fetuses were categorized into three groups determined by their gestational age (the first group was 28-31 weeks, the second group was 32-35 weeks, and the third group was 36-40 weeks).

Observation

In the present study, 81.8% of male fetuses were in the 36-40 weeks gestational age group, and 52.6 % of female fetuses were in the 32-35 weeks, gestational age group. The mean length of the spinal cords was 14.74±1.45cm, with a range of 10.95 cm to a maximum of 16.60 cm. In the full-term gestational age group, male fetuses had a greater length of spinal cord than female fetuses. Sixteen fetuses had a spinal cord termination at level L2, followed by eight fetuses at the L3 level and six fetuses at the L4 level. Out of 11 male fetuses, eight fetuses had spinal cord termination at the L2 vertebra level, two at the L3 level, and one fetus at the L4 level. In female fetuses, eight had a spinal cord termination at the L2 level, six at the L3 level, and five at the L4 level.

Conclusion

The spinal cord length and level of conus medullaris depend on the age of the fetuses. In prenatal diagnosis for different spinal cord pathology, these values can be used as reference values in future studies.

## Introduction

The spinal cord is the continuation of the brain from the lower point of the medulla and the terminal portion of the developing neural tube. During the development of the brain, which starts as a single tube, various curvatures are formed in the fore part of the neural tube. These curvatures form the forebrain and hindbrain. However, the midbrain remains in its primitive form, and the spinal cord retains its shape. The spinal cord develops within the bony vertebral canal formed by the union of individual vertebrae in the cervical, thoracic, lumbar, and sacrococcygeal regions united by intervertebral discs. The covering of the spinal cord is the same as the brain (i.e., from the inside out, the pia mater, arachnoid, and dura mater). Initially, the vertebral column and spinal cord length are the same. The growth of the vertebral column is faster than that of the spinal cord in developing fetuses, and the postnatal life spinal cord terminates early within the vertebral canal. This process is known as the ascent of the spinal cord, and this terminology has passed from one author to another [1]. The exact time of this ascent has not been determined [2]. The spinal cord generally starts to "ascend" after nine weeks, reaching the level of the last sacral piece around 12 weeks of age. The cord reaches the level of the lower border of the S1 at fifteen weeks, the lower border of the L4 at six months, and the lower border of the L3 at full term [1,3-5]. During childhood, once the spinal cord has reached its definitive level, the spinal cord and the spine's growth continues at the same rate and rhythm until adulthood [5]. The lowermost point of the spinal cord (i.e., conus medullaris) is clinically essential for anesthetists and surgeons to prevent catastrophe during anesthesia and surgery. Since the ascent of the conus medullaris continues in pre-term infants, it can be encountered at lower levels. This situation can be misinterpreted by clinicians [2].

## Materials and methods

This cross-section observational study was conducted on 30 fetuses from January 2020 to July 2022 in the Department of Anatomy, Uttar Pradesh University of Medical Science, Saifai, UP, India. The IUD (intra-uterine death) fetuses were collected from the obstetrics and gynecology departments at the same institute. The parents and University Ethical Committee gave the necessary consent for the fetus collection (Ref. no 665/UPUMS/DEAN/2019-20/EC no 2019/20 date 08-07-2019). Gestation from 28-week to 40-week fetuses was included, while fetuses with the abnormal nervous system and spinal cord development were excluded from this study.

**Figure 1:**
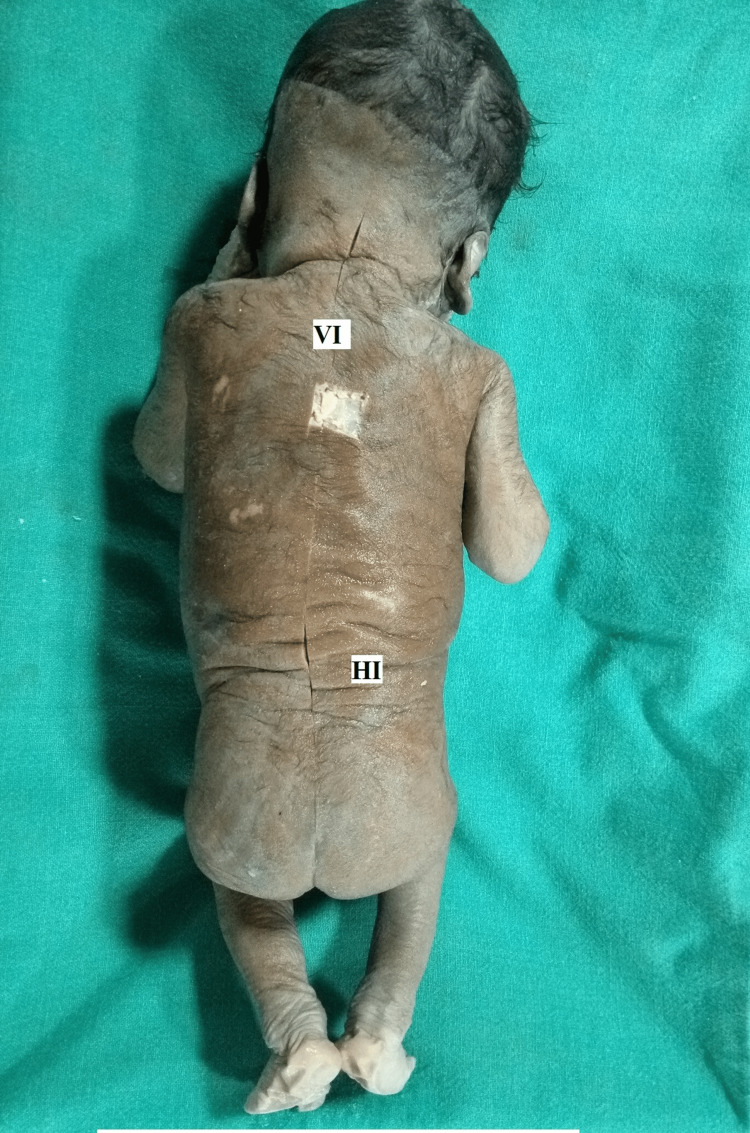
Fetus showing a line of incision (VI-Vertical line of incision and HI horizontal line of incision) of skin (S)

**Figure 2:**
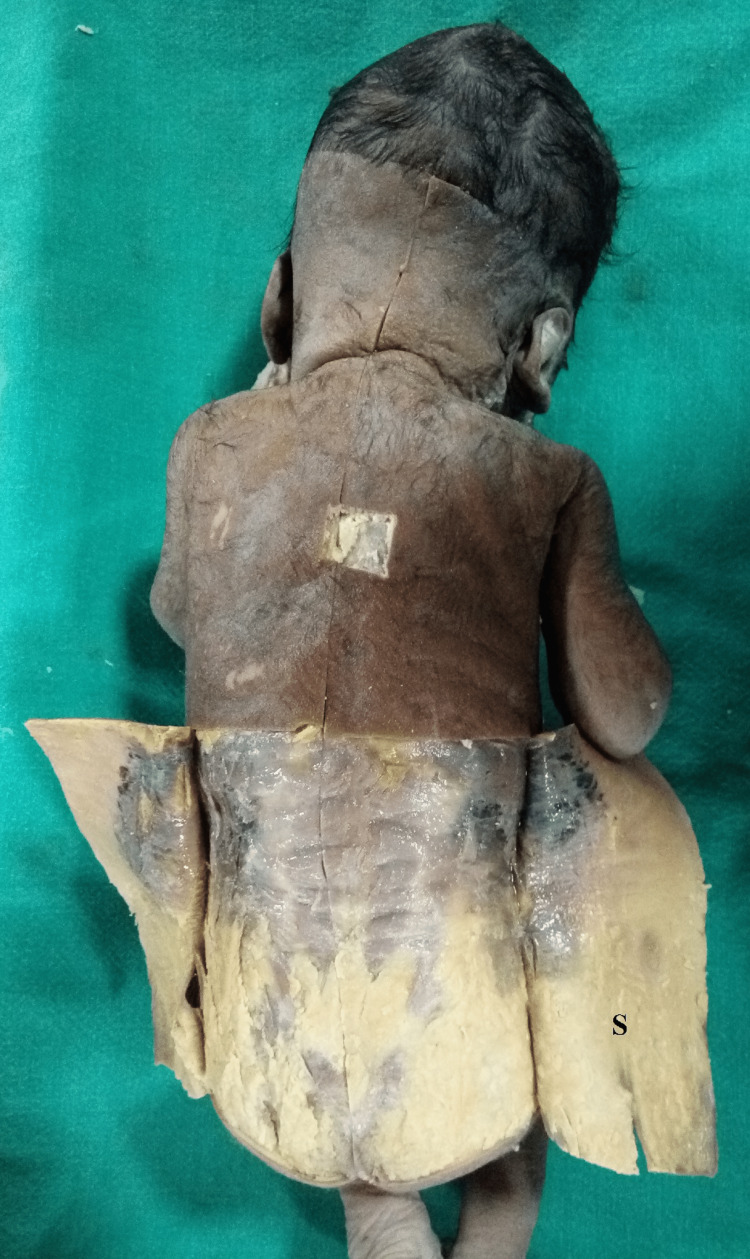
Skin (S) cut and reflected

**Figure 3:**
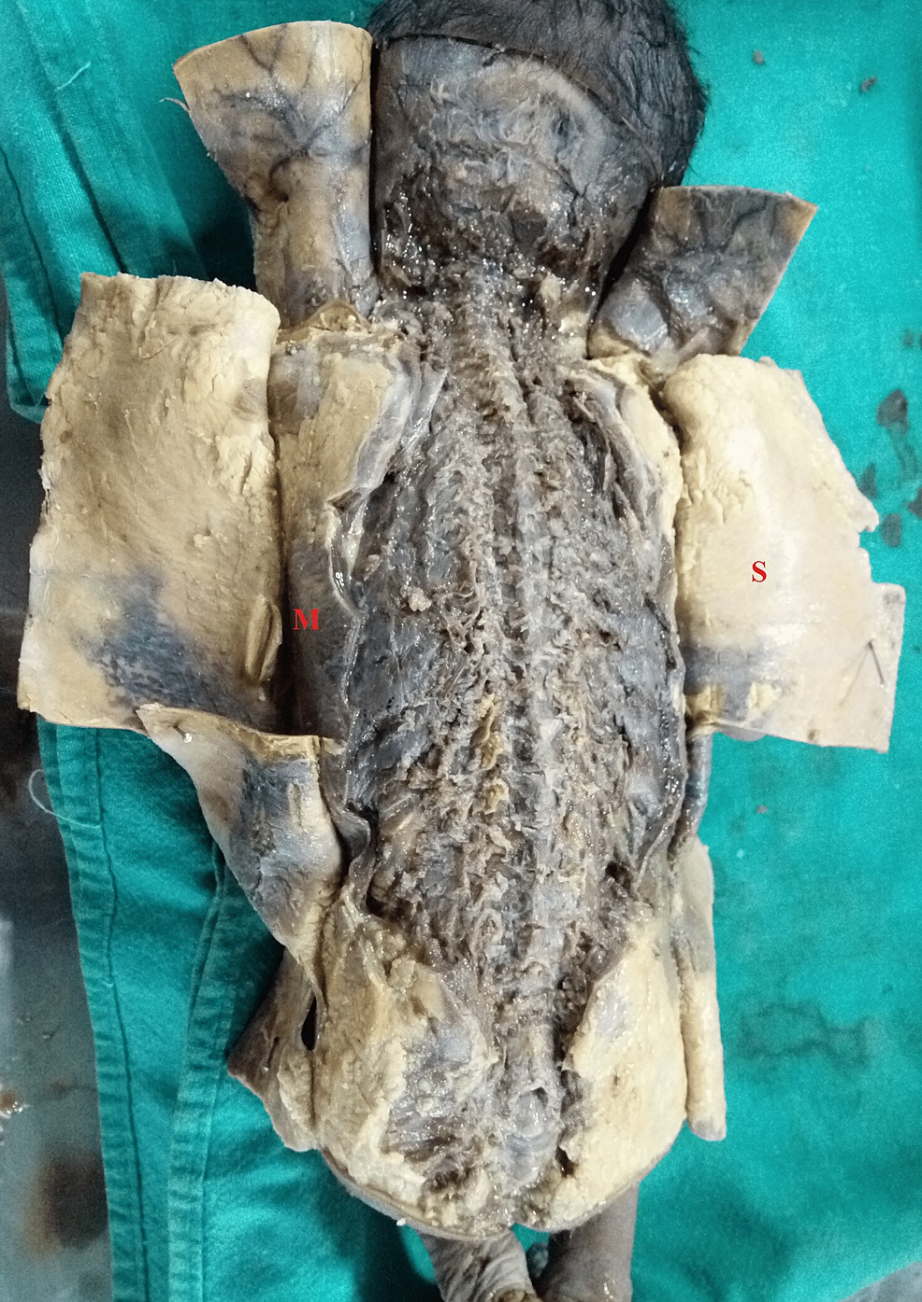
The skin (S) and muscles (M) have been cut to visualize the vertebral column

**Figure 4:**
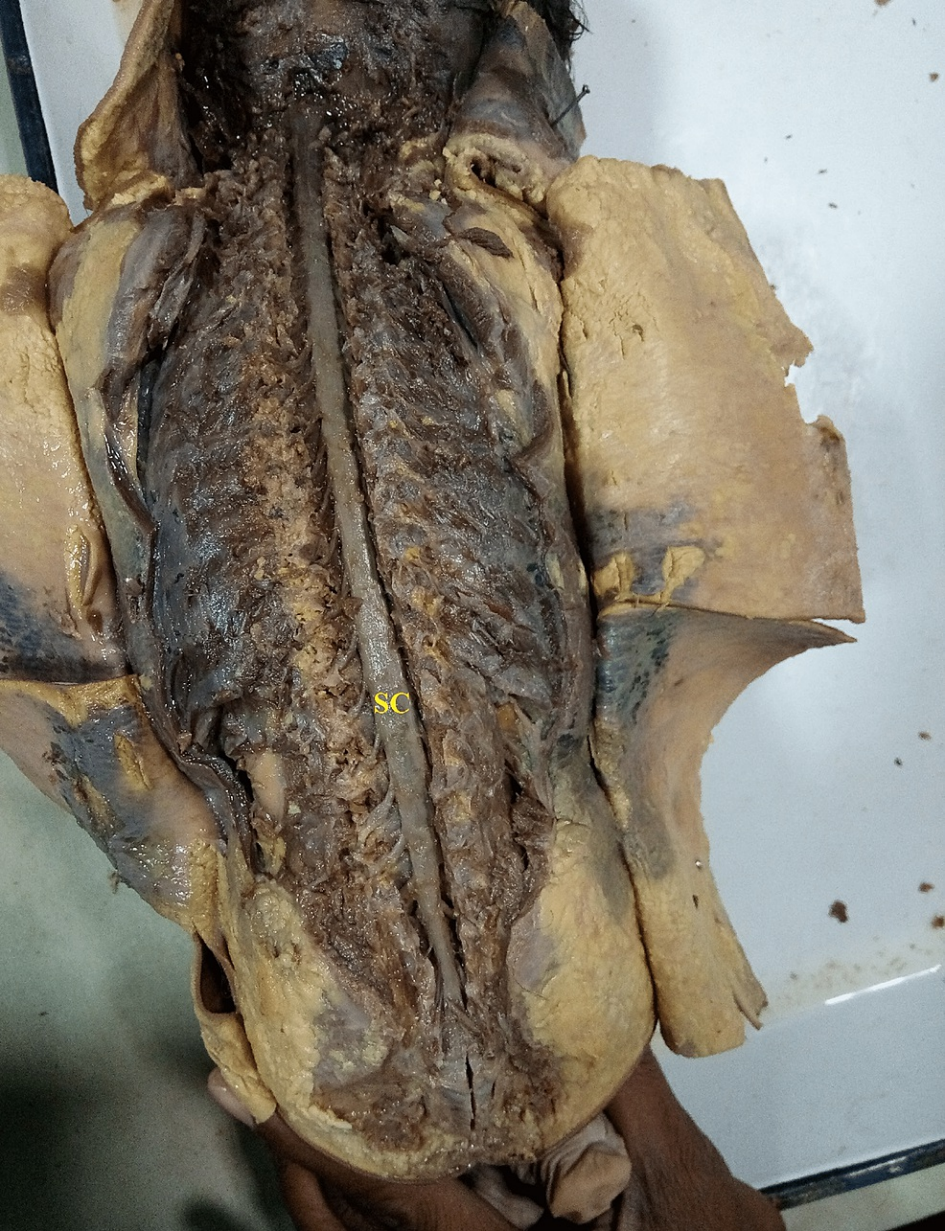
The Spine and Lamina of vertebral column has been cut and removed to show the spinal cord (SC) within its covering i.e. meninges

The early dissection includes incision of skin from the external occipital protuberance up to the lower limit of the coccyx. The skin was cut in the midline and reflected laterally. The back's superficial muscles and deep muscles from the external occipital protuberance up to the lowermost point of the sacrum and coccyx were cut and removed to visualize the vertebral column. The spines and lamina of the vertebra (laminectomy) were cut with scissors and a scalpel in the midline. The surface of the spinal cord (i.e., the dura and arachnoid mater) was clean and cut from top to bottom. All the spinal nerves coming out from the spinal cord laterally were cleaned. The meanings were cut vertically throughout their length. All the nerves of the cauda equine were separated and fanned out to visualize the complete spinal cord (Figures 1-4). 

**Figure 5:**
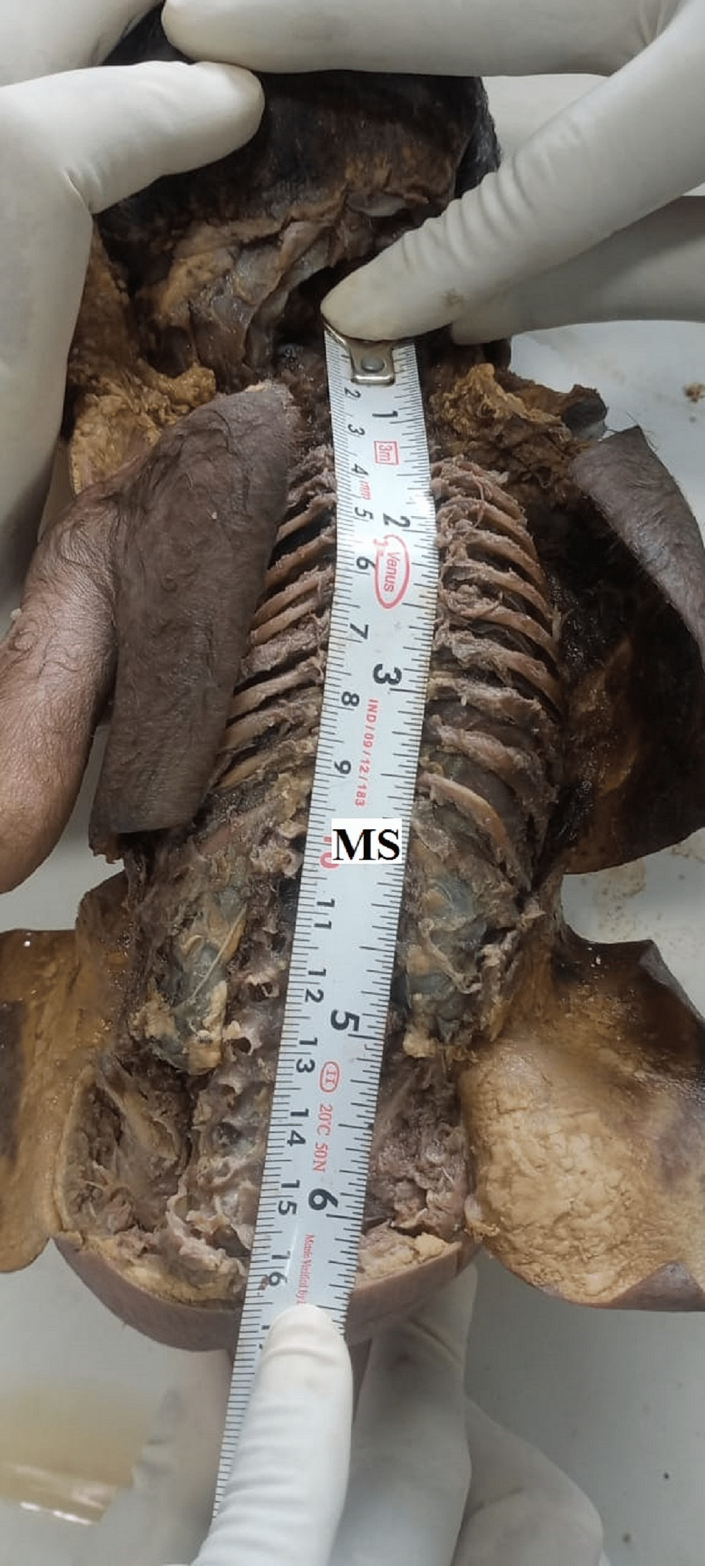
Measurement of Spinal cord within vertebral canal with the help of Measuring scale (MS)

**Figure 6:**
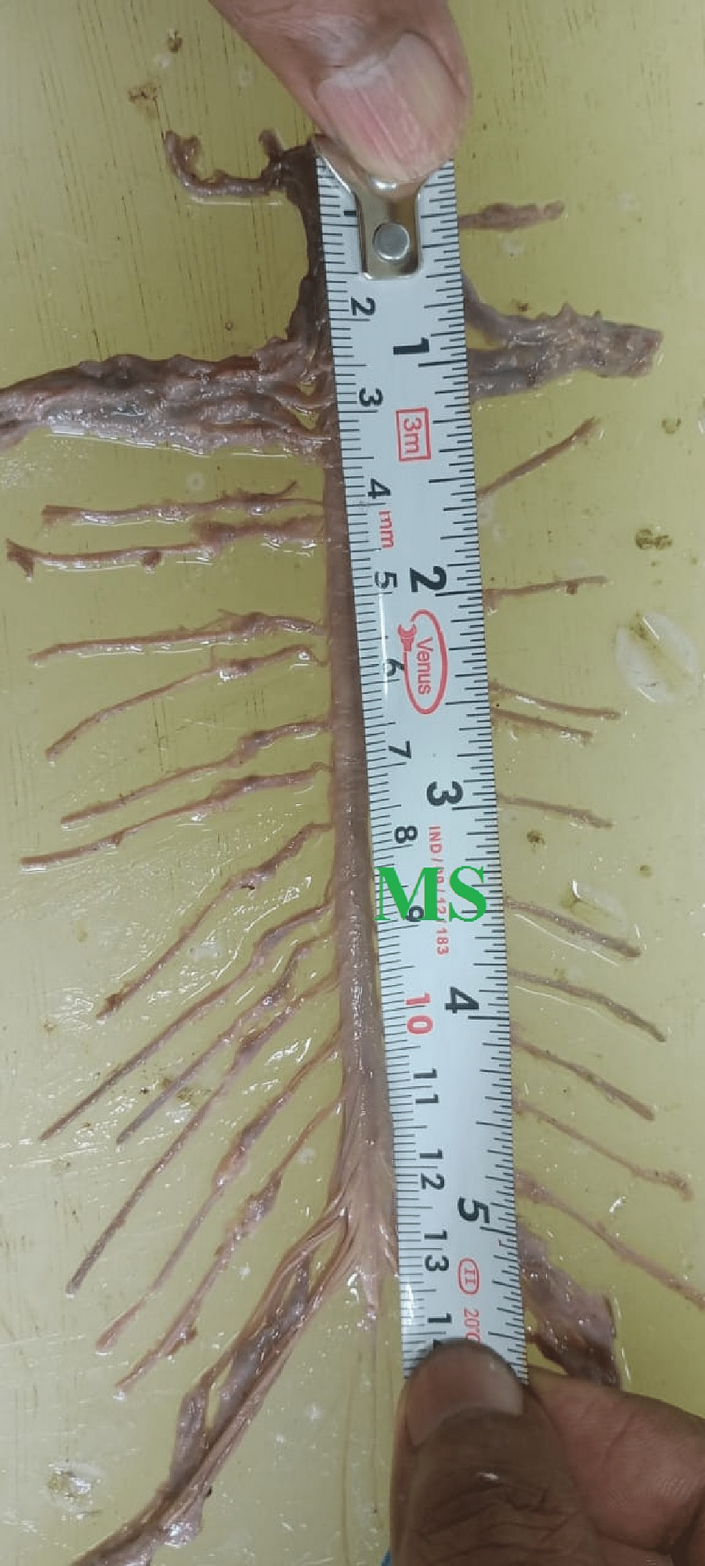
Measurement of Spinal cord outside of vertebral canal (MS- Measuring Scale).

**Figure 7:**
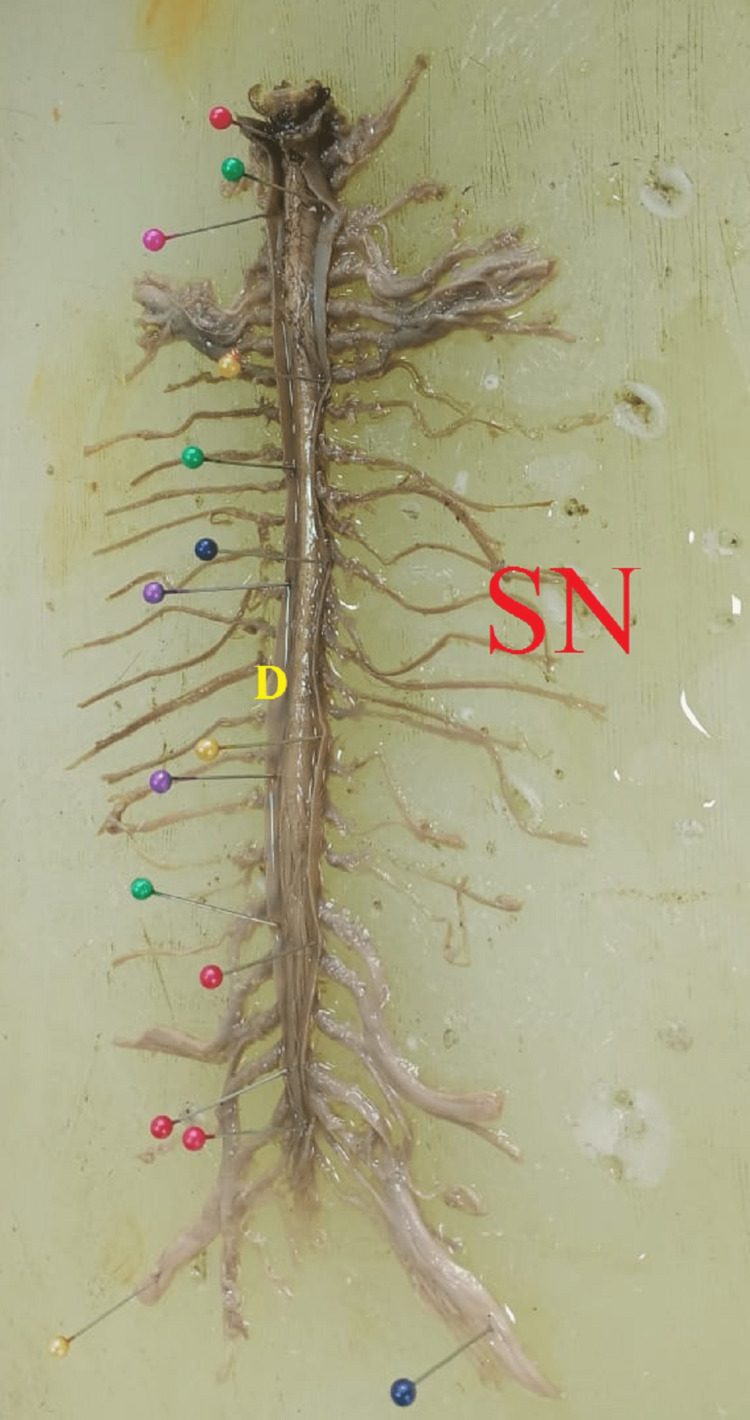
Spinal cord within dural sheath after taking out from vertebral canal (SC-Spinal Cord, SN-Spinal Nerves, D-Dura mater)

A measuring scale measured the length of the spinal cord from the uppermost point (where the first cervical nerve is coming out) to the terminal point (lowermost point of conus medullaris; Figures 5-7).

For descriptive purposes, the fetuses were categorized into three groups according to their gestational age (four fetuses in the first group of 28-31weeks, twelve fetuses in the second group 32-35weeks, and fourteen fetuses in the third group of 35-40 weeks). Data were managed on an Excel spreadsheet. In this study, we measured the length of the spinal cord within the vertebral canal and determined the lowermost point of the spinal cord within the vertebral canal.

## Results

Our study noted that the majority of 46.7% of fetuses belonged to the 36-40 weeks gestational age group, 40.0% of fetuses in the 32-35 weeks gestational age group, and 13.3% of fetuses in the 28-31 weeks gestational age group. In the present study, 63.3% of fetuses were female while 36.7% were male, and 81.8% of male fetuses came in the 36-40 weeks gestational age group while 52.6% of female fetuses were in the32-35 weeks gestational age group (Table 1).

**Table 1:** Gestational age group wise and Gender wise distributions of studied fetuses

S. No.	Gestational Age Groups (in weeks)	Total Frequency (n=30)	Male (n=11)	Female (n=19)
1	28-31	4 (13.3%)	0 (0.0%)	4 (21.1%)
2	32-35	12 (40.0%)	2 (18.2%)	10 (52.6%)
3	36-40	14 (46.07%)	9 (81.8%)	5 (26.3%)

In this study, the spinal cord lengths were 10.95 cm to 16.60 cm, and the mean cord length was 14.74±1.45 cm. The length of the spinal cord increased with increasing gestational age (see Table 2). In this study, the length of the spinal cord was greater in the males than the females in full-term gestational fetuses (Table 3).

**Table 2:** Length of Spinal cord distributions in various Gestational Age Groups

S. No.	Gestational Age Groups (in weeks)	Length of Spinal cord (cm)		
		Average	Minimum	Maximum
1	28-31	12.16±1.24	10.95	13.80
2	32-35	14.45±0.75	12.95	15.60
3	36-40	15.71±0.84	14.00	16.60
Mean		14.74±1.45	10.95	16.60

**Table 3:** Length of Spinal cord in male and female distributions in various Gestational Age Groups

Sl No.	Gestational Age Groups (in weeks)	Length of Spinal cord (Male) (cm)	Length of Spinal cord (Female) (cm)
1	28-31	--	12.16±1.24
2	32-35	13.97±1.45	14.55±0.63
3	36-40	15.94±0.81	15.30±0.82
Mean		15.59±1.17	14.24±1.39

In our study, the lowermost level of the spinal cord was noted at the L2 level in sixteen fetuses, the L3 level in eight fetuses, and the L4 level in six. In this study, in the male fetuses (n=11), the L2 level of the spinal cord was in eight fetuses, the L3 level of the spinal cord was in two fetuses, and the L4 level of the spinal cord was in one fetus. In the female (n=19) fetuses, the L2 level of the spinal cord was in eight, the L3 level of the spinal cord was in six, and the L4 level of the spinal cord was in five fetuses. We also found that the lowermost level of spinal cord termination decreases with increased gestational age; in the early third trimester (28-31 weeks), spinal cord termination was at the L4 level, whereas in the 40th week, the spinal cord was at L2 level (see Table 4).

**Table 4:** Lowermost levels of Spinal cord distributions in various Gestational Age Groups

S. No.	Gestational Age Groups (in weeks)	Lowermost level of Spinal cord			Lowermost level of Spinal cord (Male)			Lowermost of Spinal cord (Female)		
		L2	L3	L4	L2	L3	L4	L2	L3	L4
1	28-31	1	1	2	-	--	--	1	1	2
2	32-35	7	4	1	2	--	--	5	4	1
3	36-40	8	3	3	6	2	1	2	1	2
Total Frequency		16	8	6	8	2	1	8	6	5

## Discussion

The present observational cross-section study was done on 30 human fetuses of gestational ages from 28-40 weeks. Our study noted that the mean length of the spinal cord was 14.74±1.45 cm, with a minimum of 10.95 cm to a maximum of 16.60 cm. In addition, the spinal cord's length increased to increase gestational age. We also noted the gender-based difference in the spinal cord length and found that in the full-term gestational age group, the male fetuses had a longer spinal cord than the female fetuses.

In our study, the lowermost level of the spinal cord was noted at the L2 level in 16 fetuses, the L3 level in eight fetuses, and the L4 level in six fetuses, which is similar to the prospective study on the 33 fetuses by Robbin, Filly & Goldstein and suggested that termination of the conus medullaris at the L2/L3 level is considered within normal limits. They claimed that further lower-level termination beyond L3 should be considered indeterminate and require neonatal evaluation [6].

Beeket al. studied an ultrasound scan on 99 children and concluded that with the advancement of gestational age, the level of conus medullaris ascent was significant (p=0.003). The lower limit of the conus medullaris was at the L2/L3 level in fetuses of 27-29 weeks gestational age. In the fetuses of 40 weeks gestational age, the level of the conus was found to be at the L12/L2 level [7].

Sahin studied an ultrasound scan of 41 pre-perm fetuses and 64-term fetuses, and they found that in the pre-term group of fetuses, the conus medullaris level was at an L2 level of 90.4%. In the term group fetus, it was above L2 in 92.1%. The difference in the conus medullaris levels between term and pre-term neonates and genders was insignificant. They finally conclude that the ascent of the conus occurs early in fetal life [8].

Wolf et al. did an ultrasound study on 114 healthy infants to assess the time of ascent of the conus medullaris up to the normal level (L2) and found that the tip of the conus lies between the L2 and L4 level in fetuses ranging between 30-39 gestational weeks, whereas the level of the conus falls between T12/L1 to L1/L2 in 40-63 weeks [9].

Sun et al. suggested using an MRI as an accurate and useful method to determine the level of conus medullaris in term infants. They concluded that the conus medullaris reaches the normal adult level at birth and does not ascend afterward [10].

Albert-Neels et al. studied newborn infants' cadavers with a sagittal MRI of children, adolescents, and young adults. The authors found a significant difference in newborn infants from other higher groups. In infants, the lower limit of the conus was found at the L2/L3 level (16%). In contrast, in the childhood stage, adolescent population, and young adult group, it was at the levels of T12/L1 and the lower third of L1 (21%), the middle third of L1, and L1/L2 (19%), and level of L1/L2 (25%), respectively. They found the difference in the conus termination in infants within one year and older than one year. In this study, the spinal cord termination was not found caudal to the L3 vertebral body [11]. Nakashima et al. studied 629 individuals from all age groups and sexes. They concluded that most individuals (92.2%) have conus termination at the T12-L1 level, whereas 7.8% have the conus cranial at the T12 level. The authors found that the caudal levels of the conus were significantly associated with lower height and a smaller pelvic index, thus further determining a gendered difference associated with the terminal level of conus medullaris [12]. The work of Gatonga, Ogeng'o, and Awori on 112 cadavers revealed the lower limit of conus medullaris at or below the upper one-third of the L2 level [13]. Vettivel found the lower border of the spinal cord at the second lumbar vertebra (L2) [14].

Rao reported his findings on 19 South Indian fetuses and one newborn [15]. He reported that the adult level of spinal cord termination was reached in some fetuses as early as the 214 mm crown-rump length (CR length) stage. The recession rate of the spinal cord and ascent of the conus medullaris also seemed to differ in the North Indian and South Indian groups.

We further noted that the lowermost level of the spinal cord termination at the L2 level increased in the gestational age group in the 40th week. It was increasingly at the L4 level in the early third trimester (28-31 weeks). Mottet et al. reported that an L4 level or greater was reached at the gestational age of 13-18 weeks from L1 to L2 at approximately 40 weeks [16]. Levins reported that the spinal cord extends to the L1-2 disc in 51% of people and the L2-3 disc or below in 12% [17]. Boon et al. reported that only 25% of the cords they studied ended below the L1-2 disc [18], whereas Reimann and Anson found that 49% of the cords they studied were below this level [19]. Similarly, Broadbent et al. reported that 19% of the spinal cord terminations were below the L1 in a series of 100 patients undergoing spinal MRI scans [20]. Saifuddin et al. reported that only six cords out of 504 (1.19%) terminated below the L2-3 disc [21].

Boon et al. stated that the difficulty in determining spinal cord termination (CMT) might be due to the presence of lumbosacral transitional vertebrae in 8-15% of subjects [18].

We further noted that out of 11 male fetuses, eight were at the L2 level, two were at the L3 level, and only one was at the L4 level; while in females, eight fetuses were at the L2 level, six fetuses at L3 level, and five fetuses were at L4 level. According to Thomson (1894) and Needles (1935), the adult female spinal cord terminates at a lower level than that of the male [22]. Vettivel did not comment on sex differences in the fetuses and neonates [14]. Therefore, not much difference can be detected between the sexes from their tables. However, a tendency in adult females for the spinal cord to terminate at lower levels than in adult males was reported by Vettivel, Reimann, and Anson [14,19]. If there is a difference, then it is possible that, at least in the earlier and later stages of development, the spinal cord in the female terminates at a slightly higher level than in the male, and all the female neonates have the spinal cords terminating at the L1 level.

## Conclusions

In this study, using distinct anatomic landmarks to prevent bias, the ascent of the conus medullaris occurred throughout the gestational age. A follow-up scan and proper evaluation are required to prevent catastrophe for fetuses whose spinal cord termination levels were abnormally low (lower than the L3 to L4 levels). Early postnatal treatment through a multidisciplinary approach is recommended for patients with abnormally low-level spinal cords (e.g., pediatric surgery, neurosurgery).
